# Decompensation in Critical Care: Early Prediction of Acute Heart Failure Onset

**DOI:** 10.2196/19892

**Published:** 2020-08-07

**Authors:** Patrick Essay, Baran Balkan, Vignesh Subbian

**Affiliations:** 1 College of Engineering The University of Arizona Tucson, AZ United States; 2 Department of Systems and Industrial Engineering Department of Biomedical Engineering The University of Arizona Tucson, AZ United States

**Keywords:** critical care, heart failure, intensive care units, machine learning, time series, heart, cardiology, prediction, chronic disease, ICU, intensive care unit

## Abstract

**Background:**

Heart failure is a leading cause of mortality and morbidity worldwide. Acute heart failure, broadly defined as rapid onset of new or worsening signs and symptoms of heart failure, often requires hospitalization and admission to the intensive care unit (ICU). This acute condition is highly heterogeneous and less well-understood as compared to chronic heart failure. The ICU, through detailed and continuously monitored patient data, provides an opportunity to retrospectively analyze decompensation and heart failure to evaluate physiological states and patient outcomes.

**Objective:**

The goal of this study is to examine the prevalence of cardiovascular risk factors among those admitted to ICUs and to evaluate combinations of clinical features that are predictive of decompensation events, such as the onset of acute heart failure, using machine learning techniques. To accomplish this objective, we leveraged tele-ICU data from over 200 hospitals across the United States.

**Methods:**

We evaluated the feasibility of predicting decompensation soon after ICU admission for 26,534 patients admitted without a history of heart failure with specific heart failure risk factors (ie, coronary artery disease, hypertension, and myocardial infarction) and 96,350 patients admitted without risk factors using remotely monitored laboratory, vital signs, and discrete physiological measurements. Multivariate logistic regression and random forest models were applied to predict decompensation and highlight important features from combinations of model inputs from dissimilar data.

**Results:**

The most prevalent risk factor in our data set was hypertension, although most patients diagnosed with heart failure were admitted to the ICU without a risk factor. The highest heart failure prediction accuracy was 0.951, and the highest area under the receiver operating characteristic curve was 0.9503 with random forest and combined vital signs, laboratory values, and discrete physiological measurements. Random forest feature importance also highlighted combinations of several discrete physiological features and laboratory measures as most indicative of decompensation. Timeline analysis of aggregate vital signs revealed a point of diminishing returns where additional vital signs data did not continue to improve results.

**Conclusions:**

Heart failure risk factors are common in tele-ICU data, although most patients that are diagnosed with heart failure later in an ICU stay presented without risk factors making a prediction of decompensation critical. Decompensation was predicted with reasonable accuracy using tele-ICU data, and optimal data extraction for time series vital signs data was identified near a 200-minute window size. Overall, results suggest combinations of laboratory measurements and vital signs are viable for early and continuous prediction of patient decompensation.

## Introduction

### Background

Intensive care units (ICUs) are data-rich clinical environments involving complex decision-making for patients who are critically ill making them a major area of health care innovation [1]. The ability to continuously monitor patients in the ICU provides unique opportunities for analytics such as estimation of physiological states and prediction of decompensation (ie, clinical deterioration) or patient outcomes [2]. There has been substantial progress in terms of predicting longer-term outcomes such as mortality and readmission rates in patients with heart failure, but there is limited work around predicting shorter-term clinical events in the ICU, such as acute heart failure onset [3-5]. Predicting such decompensation events allows for prevention and mitigation steps while patients are in the ICU and promotes a proactive decision-making process for clinicians, potentially resulting in timely interventions and improved patient outcomes.

In this work, we present the application of machine learning techniques for predicting decompensation in critical care settings using acute heart failure onset as the prediction outcome [6]. The objectives of this study are to examine the prevalence of three heart failure risk factors (ie, coronary artery disease, hypertension, or myocardial infarction); to apply and evaluate machine learning techniques to predict heart failure onset in patients with and without one of the three known risk factors; and to evaluate features of interest including aggregate time series vital signs data, laboratory values, and other physiological inputs used in traditional clinical scoring systems.

Heart failure is a major cause of mortality and morbidity worldwide, and a major public health concern. It is a complex clinical syndrome where cardiac dysfunction impairs the ability of the ventricle to fill and eject blood, leading to a wide range of signs and symptoms and unspecific diagnosis [7-9]. Although there have been advances in therapies, further understanding of prognosis and management of acute heart failure is needed [10]. This is particularly true in critical care where heart failure may be of secondary concern to clinicians relative to primary ICU diagnosis.

There has been interest in shifting prognostication of decompensation events such as onset of heart failure to a remote monitoring team (tele-ICU) [11]. Although such telemedicine-based efforts have become increasingly common in cardiovascular ICUs, risk of acute heart failure onset has not been extensively investigated through a machine learning and tele-ICU lens [12]. Additionally, there are several known risk factors of heart failure, including hypertension, coronary artery disease, myocardial infarction, obesity, diabetes, and other lifestyle factors such as alcohol intake, smoking, and leisure activity [13]. Of these, hypertension, coronary artery disease, and myocardial infarction are identifiable key risk factors of acute heart failure and relevant to remote ICU monitoring.

### Significance

Multiple prior studies related to heart failure in different settings (eg, inpatient vs outpatient) using dissimilar data sources (eg, home-based monitoring data vs in-hospital clinical data) have been conducted [14,15]. These studies used features such as change in body weight, heart rate, and blood pressure under the hypothesis that hemodynamic changes in patients can be characterized in continuous physiological data collected by the patient at home. In critical care settings, many of the variables used by the bedside clinical team are readily available to the remote tele-ICU team as well for deeper analytics.

Previous studies have modeled risk of hospitalization, long-term survival rates, and mode of death prediction as a result of heart failure [16-18]. Models used features related to clinical status, therapy, and laboratory parameters including home-based physiological telemonitoring [19]. Generally, these studies use temporal data to make longer-term (ie, months to years) predictions [20].

These and other studies illustrate potential and previous accomplishments in heart failure prediction, but to our knowledge, models have not been developed in the context of critical care and the fast-paced ICU environment or used the expansive capabilities of tele-ICU data. These previous studies do, however, suggest that trends in patient physiology and hemodynamics may be leveraged for early heart failure prediction.

Our study attempts to predict onset of acute heart failure by examining readily available physiological discrete and time series data on a truncated scale near the time of ICU admission. We applied data extraction methods similar to approaches used in longer-term prediction models and comparable physiological measurements, in addition to potentially more extensive and reliable tele-ICU data as compared to home-based measurements.

## Methods

### Data Source and Preprocessing

In this study, we used the eICU Collaborative Research Database [21], which contains remotely monitored critical care data from adult patients admitted to over 200 hospitals in the United States from 2014-2015 [22]. The database includes basic patient characteristics as well as medications, laboratory values, vital signs, and other discrete physiological variables measured at the bedside ICU and interfaced with the tele-ICU. We selected both multivariate logistic regression and decision tree models for predicting acute heart failure, given their interpretable nature.

Patient ICU stays were extracted based on primary admission diagnosis and subsequent diagnostic codes during the same unit stay. Inclusion criteria were such that each ICU stay must not have a primary admission diagnosis of heart failure (ie, the patient was admitted to the ICU for a reason other than heart failure). Readmissions were included unless the subsequent stays were primarily due to heart failure.

Patient stays were segregated based on three heart failure risk factors: coronary artery disease, hypertension, and myocardial infarction. In each risk factor group, patients were categorized by heart failure onset after primary admission diagnosis. A fourth group of *nonrisk factor patients* was extracted including all patients admitted for reasons other than heart failure and did not have record of one of the three risk factors. The International Classification of Diseases version 9 (ICD-9) codes were used to determine heart failure and risk factors (Table 1).

**Table 1:** Heart failure ICD-9 codes for cohort discovery.

ICD-9^a^ code		Description
**Heart failure**		
	398.91	Rheumatic heart failure (congestive)
	428.0	Congestive heart failure, unspecified
	428.1	Left heart failure
	428.20	Systolic heart failure, unspecified
	428.21	Acute systolic heart failure
	428.22	Chronic systolic heart failure
	428.23	Acute on chronic systolic heart failure
	428.30	Diastolic heart failure, unspecified
	428.31	Acute diastolic heart failure
	428.32	Chronic diastolic heart failure
	428.33	Acute on chronic diastolic heart failure
	428.40	Combined systolic and diastolic heart failure, unspecified
	428.41	Acute combined systolic and diastolic heart failure
	428.42	Chronic combined systolic and diastolic heart failure
	428.43	Acute on chronic combined systolic and diastolic heart failure
	428.9	Heart failure, unspecified
**Coronary Artery Disease**		
	414.0	Coronary atherosclerosis
**Hypertension^b^**		
	401	Essential hypertension
	402.00	Malignant hypertensive heart disease without heart failure
	402.10	Benign hypertensive heart disease without heart failure
	402.90	Unspecified hypertensive heart disease without heart failure
**Myocardial Infarction**		
	410	Acute myocardial infarction
	412	Old myocardial infarction

^a^ICD-9: International Classification of Diseases version 9.

^b^ICD-9 codes for hypertensive conditions *with* heart failure were not included because heart failure onset later in the intensive care unit stay is used as the prediction outcome.

Vital signs, laboratory values, and Acute Physiology and Chronic Health Evaluation (APACHE) IVa variables were extracted for all four patient groups (three risk factor groups and the *nonrisk factor* patients). APACHE variables included features such as age and gender, admission diagnoses, and worst physiological values in the first 24 hours of ICU admission (eg, white blood count, temperature, respiratory rate) [23]. In total, 35 APACHE variables were extracted for each patient stay. Discrete APACHE variables such as *admission diagnosis* and *admission source* that do not reflect an ordinal or hierarchical relationship were encoded using the one-hot vector method.

Laboratory variables were selected based on those measurements that are routinely performed under normal ICU operations. We found overlap with our extracted lab values and those used in previous studies to predict heart failure [24]. In total, we used seven lab measurements: bedside glucose, potassium, sodium, glucose, hemoglobin, creatinine, and blood urea nitrogen. All of which were within the ten most frequently performed laboratory measurements in our data set. To predict decompensation as early in the ICU as possible, only the first measurement for each of the selected lab values was retained for model input.

Vital signs included data collected at both regular and irregular intervals. For example, temperature, heart rate, and respiratory rate tend to be regularly recorded in clinical practice and subsequently archived to the database, while cardiac output and noninvasive blood pressure may be recorded at irregular time intervals. When available at the bedside, vital signs data are collected from bedside monitoring devices at a frequency of 1-minute averages and archived as 5-minute median values. A total of 23 physiological vital signs features were extracted and are listed in Multimedia Appendix 1.

**Figure 1:**
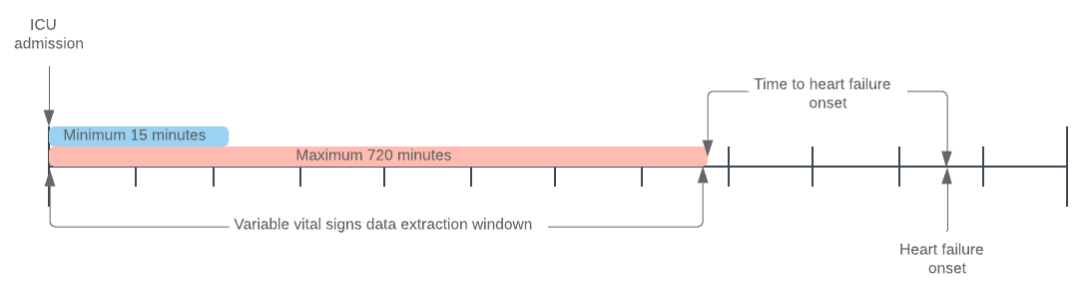
Timeline illustrating vital signs data extraction window from the time of ICU admission. ICU: intensive care unit.

To predict heart failure onset as early as possible, vital signs were extracted at variable time windows based on number of minutes from ICU admission (Figure 1). For example, a time window of 180 minutes results in vital signs extraction from the time of ICU admission to 180 minutes after admission. The extraction window was varied from 15 minutes to 720 minutes (12 hours) from the time of admission. All available vital signs data were aggregated to mean, median, minimum, maximum, and standard deviation for each feature. This eliminated variations in the time series length between unit stays caused by irregular data sampling and missing data within each series.

### Multivariate Logistic Regression

We applied multivariate logistic regression using a binary L2 penalized minimization cost function where the target class prediction (*ŷ*) is a linear combination of the input features with a coefficient vector *w* = (*w*_1_, ..., *w_p_*) and intercept *w*_0_ (1), where input vectors *x* = (*x*_1_, ..., *x_p_*) consist of discrete physiological variables and aggregate vital signs measurements.

*ŷ*(w*,x*) = *w*_0_ + *w*_1_*x*_1_ + ... + *w_p_x_p_***(1)**

Model input features minimize the cost variable (*c*) and coefficients (*w*) in the minimization cost function (2).


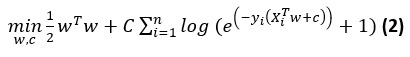


Combinations of input variables were tested for each *risk factor* and *nonrisk factor* cohort.

### Random Forest

The random forest model was applied with the Gini impurity measure for each cohort and compared to logistic regression performance. Random forest is an ensemble method that uses a collection of tree-structured classifiers to calculate the average prediction over all individual decision tree classifiers. Inputs to each tree consist of randomly split combinations of input feature vectors *x_p_* ∈ *R^n^*, *i* = 1, …, *l* and target labels (heart failure or not heart failure) *y* ∈ *R^l^*. The data (*Q*) at each node (*m*) was used to calculate Gini impurity by multiplying node importance by *H*(*X_m_*) through (3), where *θ* = (*j*, *t_m_*) for each data split consisting of a feature *j* and threshold *t_m_*. Node importance was denoted as *n_left or right_*, and the equation is recursed for each node subset until the maximum depth is reached (ie, *N_m_*<*min_samples_* or *N_m_*=1).


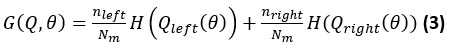


A minimum split requirement of two samples was used with no maximum depth parameter, meaning all tree nodes were expanded until leaves contained less than two samples. The maximum number of estimators (number of trees in the forest) was chosen empirically during testing and held constant at 150 estimators for all input combinations.

### Test and Evaluation

All model input variables were standardized centering the data around zero by subtracting the mean of each feature and dividing by the standard deviation. Model inputs consisted of lab values, APACHE variables, or aggregate vital signs as individual sets of inputs or as combinations of input features (ie, labs and vitals, labs and APACHE, vitals and APACHE, all three input data types). Each logistic regression and random forest model was tested with each data type and combination of inputs.

More extensive testing was performed using vital signs only as the data extraction window was varied to determine the impact of aggregating longer time series. Vital signs inputs were tested from the minimum to maximum data extraction window (15-720 minutes from ICU admission).

We then used the random forest model to identify the most important input features for predicting heart failure. The ensemble tree structure of random forest is easily interpretable and allows for the calculation of the relative importance of each feature.

Model performance was evaluated across all four patient cohorts. In addition, we combined coronary artery disease, hypertension, and patients with myocardial infarction into a single *risk factor* cohort for side-by-side comparison with the *nonrisk factor* patients. Results are included for individual patient groups and the combined *risk factor patients*.

Training and testing were performed with 67% train and 33% test split allowing for a sufficient number of patients to return statistically meaningful results and a test group which was representative of each cohort as a whole. Model performance was evaluated by accuracy and area under the receiver operating characteristic curve (AUC). Precision (true positives divided by the sum of true positives and false positives) and recall (true positives divided by the sum of true positives and false negatives) are also calculated along with precision-recall (P-R) curves to describe how good the models are at predicting heart failure correctly as opposed to correctly predicting patients with nonheart failure. Data preprocessing and prediction modeling was performed in Python (v.2.7.14; Python Software Foundation) using the Pandas (v.0.23.4) [25], Seaborn (v.0.9.0) [26], and sci-kit learn package (v.0.19) [27] libraries.

## Results

Our study sample consisted of 145,913 adult ICU stays from 122,884 unique patients with a slightly higher number of male than female patients covering a wide range of diagnoses. Additional patient characteristics within each risk factor cohort and *nonrisk factor patients* are shown in Table 2.

**Table 2:** Heart failure and nonheart failure patient characteristics.

Risk factor cohort		Coronary artery disease	Hypertension	Myocardial infarction	Nonrisk patients
Patients, n		2885	17,376	6273	96,350
ICU^a^ stays, n		3161	19,424	6689	116,639
Readmissions, n (%)		276 (8.73)	2048 (10.54)	416 (6.22)	20,289 (17.39)
Heart failure rate, n (%)		715 (22.62)	3058 (15.74)	799 (11.95)	7571 (6.49)
Age (years), median (IQR)		71 (16)	67 (21)	66 (20)	64 (24)
Gender (male), n (%)		2154 (68.14)	10,304 (53.04)	4255 (63.61)	62,387 (53.49)
**Ethnicity, n (%)**					
	Caucasian	2605 (82.41)	13,161 (67.76)	5366 (80.22)	91,176 (78.17)
	African American	263 (8.32)	3333 (17.16)	533 (7.97)	12,461 (10.68)
	Hispanic	137 (4.33)	1549 (7.97)	196 (2.93)	3817 (3.27)
	Asian	21 (0.66)	333 (1.71)	91 (1.36)	1628 (1.40)
	Native American	11 (0.35)	69 (0.36)	21 (0.31)	926 (0.79)
	Other/unknown	124 (3.93)	979 (5.04)	482 (7.20)	1426 (5.68)
APACHE^b^ score, median (IQR)		54 (29)	50 (28)	46 (30)	51 (32)
ICU LOS^c^ (days), median (IQR)		1.99 (2.69)	1.86 (2.51)	1.69 (2.06)	1.80 (2.29)
ICU mortality, n (%)		146 (4.62)	737 (3.79)	432 (6.46)	7127 (6.11)
Hospital LOS (days), median (IQR)		6.32 (7.39)	5.43 (6.99)	3.86 (5.86)	5.61 (7.06)
Hospital mortality, n (%)		245 (7.75)	1319 (6.79)	632 (9.45)	11,255 (9.65)

^a^ICU: intensive care unit.

^b^APACHE: Acute Physiology and Chronic Health Evaluation.

^c^LOS: length of stay.

Patients with hypertension were much more prevalent than patients with myocardial infarction or coronary artery disease, as might be expected. Coronary artery disease, hypertension, and myocardial infarction account for a total of 4572 (37.65%) of 12,143 total heart failure unit stays, suggesting that most patients present to the ICU without diagnosis of one of these three risk factors. It is important to note, however, that we are examining remote monitoring critical care data only. Risk factors may be captured in hospital bedside records prior to ICU admission. Readmissions to the ICU for illnesses other than heart failure account for 2740 of 29,274 (9.36%) ICU stays in the three risk factor cohorts and 20,289 of 116,639 (17.39%) stays of *nonrisk factor patients*.

**Figure 2:**
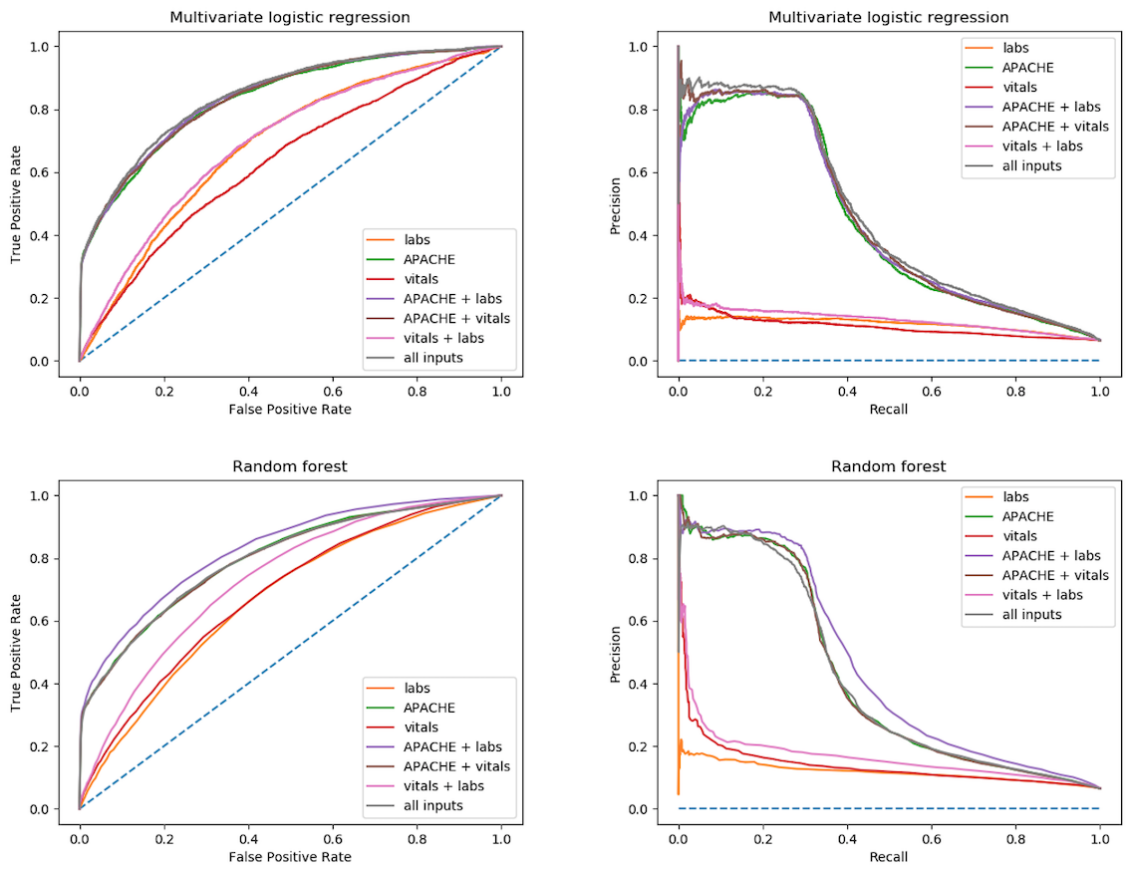
Nonrisk factor patients (patients presenting to the intensive care unit without risk factor of heart failure) area under receiver operating characteristic curve and precision-recall curve for both multivariate logistic regression and random forest models. Each curve represents a different model input combination. Vital signs data extraction window was held constant at 360 minutes for all inputs. APACHE: Acute Physiology and Chronic Health Evaluation.

**Figure 3:**
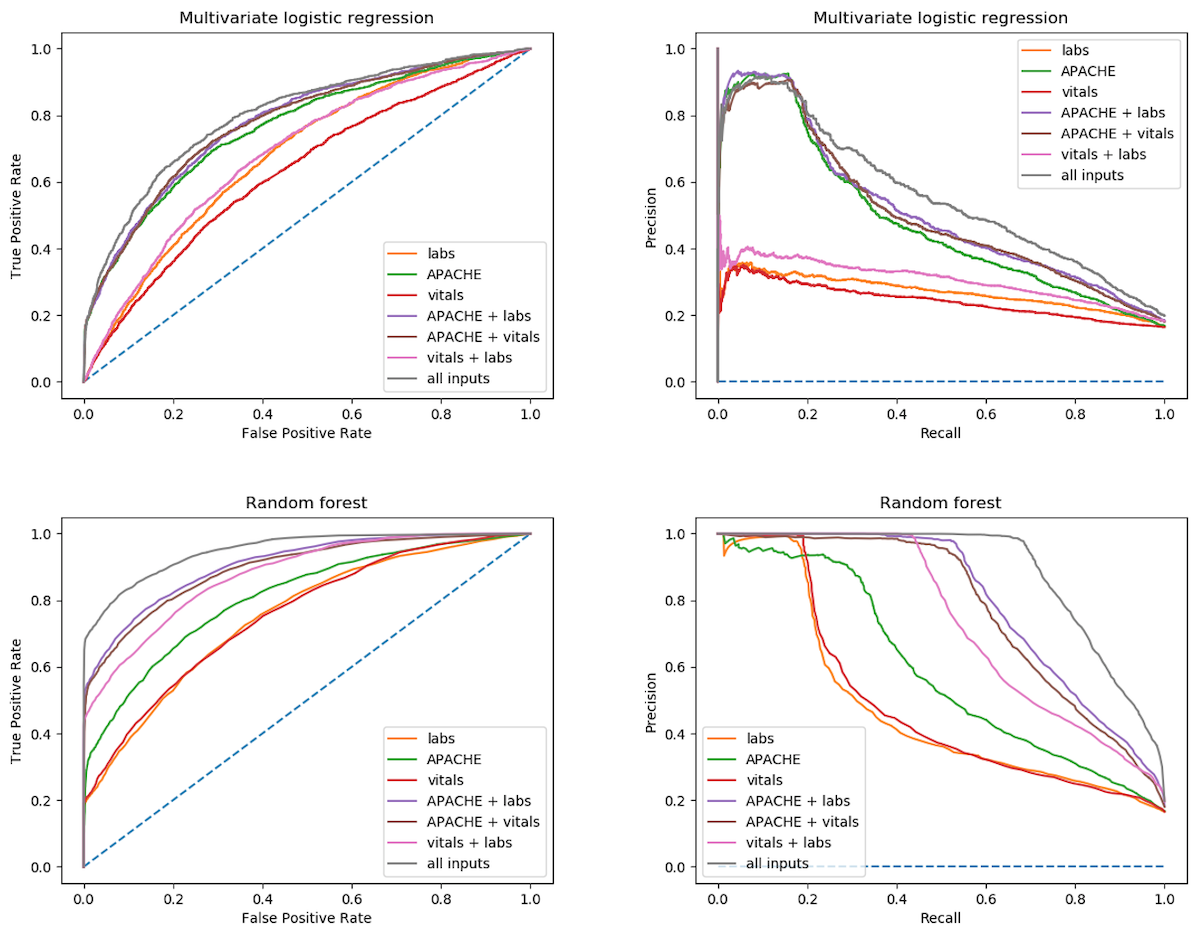
Risk factor patients (patients presenting to the intensive care unit with coronary artery disease, hypertension, or myocardial infarction) area under receiver operating characteristic curve and precision-recall curve for both multivariate logistic regression and random forest models. Each curve represents a different model input combination. The vital signs data extraction window was held constant at 360 minutes for all inputs. APACHE: Acute Physiology and Chronic Health Evaluation.

The AUC and P-R curves for the *risk factor* and *nonrisk factor patients* for both logistic regression and random forest are shown in Figures 2 and 3. Additional AUC and P-R curves for each risk factor group individually are included in Multimedia Appendix 2. For all AUC and P-R curves, the vital signs data extraction window was held constant at 360 minutes from ICU admission. Clearly, discrete APACHE variables outperform lab values and vital signs individually; however, combining inputs with APACHE variables improves results. Additionally, it appears lab values had a greater impact on performance than vital signs alone as seen by the “APACHE + labs” curves relative to other combinations of input variables.

**Table 3:** Logistic regression and random forest F1 scores across model input combinations. Vital signs data extraction window held constant at 360 minutes for all trials.

Patients		Logistic Regression	Random Forest
**Risk factor patients**			
	APACHE^a^	0.82	0.85
	Labs	0.76	0.82
	Vitals	0.76	0.83
	APACHE + labs	0.81	0.90
	APACHE +vitals^b^	0.81	0.90
	Labs + vitals	0.75	0.88
	APACHE + labs + vitals	0.81	0.93
**Nonrisk factor patients**			
	APACHE	0.94	0.94
	Labs	0.90	0.90
	Vitals	0.90	0.90
	APACHE + labs	0.94	0.94
	APACHE +vitals	0.94	0.94
	Labs + vitals	0.90	0.90
	APACHE + labs + vitals	0.94	0.94

^a^APACHE: Acute Physiology and Chronic Health Evaluation.

^b^Vital signs extraction window of 360 minutes from intensive care unit admission.

Both models were compared across input combinations for *risk factor* and *nonrisk factor patients* using the F1 score (Table 3). Interestingly, logistic regression with APACHE and labs inputs had the highest F1 score, while, in general, random forest has higher AUC, accuracy, and weighted average precision and recall (Tables 4 and 5). In this application, precision shows what proportion of heart failure identifications were actually heart failure, and recall is the proportion of heart failure stays that were correctly identified [28]. Random forest with APACHE, laboratory measurements, and vital signs combined model inputs had the highest performance metrics at an AUC of 0.9503, accuracy of 93.15%, and micro- and macroweighted average precision and recall of 0.93 and 0.93, respectively. It is important to note that, although the weighted average precision and recall are fairly high, the P-R curves exhibit a steep drop in precision as recall increases.

**Table 4:** Heart failure prediction accuracy and AUC.

Models		Risk factor patients		Nonrisk factor patients	
		AUC^a^	Accuracy	AUC	Accuracy
**Logistic regression**					
	APACHE^b^ + labs	0.7790	0.8417	0.8396	0.9501
	APACHE + vitals^c^	0.7775	0.8456	0.8374	0.9512
	Labs + vitals^c^	0.6859	0.8125	0.6947	0.9333
	APACHE + labs + vitals^c^	0.8005	0.8357	0.8458	0.9502
**Random forest**					
	APACHE + labs	0.9081	0.9112	0.8285	0.9499
	APACHE +vitals^c^	0.8956	0.9080	0.7967	0.9488
	Labs + vitals^c^	0.8794	0.8965	0.7318	0.9343
	APACHE + labs + vitals^c^	0.9503	0.9315	0.7999	0.9471

^a^AUC: area under the receiver operating characteristic curve.

^b^APACHE: Acute Physiology and Chronic Health Evaluation.

^c^Vital signs extraction window of 360 minutes from intensive care unit admission.

**Table 5:** Logistic regression and random forest precision and recall.

Models		Risk factor patients		Nonrisk factor patients	
		Precision^a^	Recall^b^	Precision^a^	Recall^b^
**Logistic regression**					
	APACHE^c^ + labs	0.82	0.84	0.94	0.95
	APACHE +vitals^d^	0.83	0.85	0.95	0.95
	Labs + vitals^d^	0.74	0.81	0.89	0.93
	APACHE + labs + vitals^d^	0.82	0.84	0.95	0.95
**Random forest**					
	APACHE + labs	0.92	0.91	0.95	0.95
	APACHE +vitals^d^	0.91	0.91	0.94	0.95
	Labs + vitals^d^	0.91	0.90	0.92	0.93
	APACHE + labs + vitals^d^	0.93	0.93	0.94	0.95

^a^Weighted average microprecision and macroprecision.

^b^Weighted average microrecall and macrorecall.

^c^APACHE: Acute Physiology and Chronic Health Evaluation.

^d^Vital signs model inputs at 360 minutes from intensive care unit admission.

**Figure 4:**
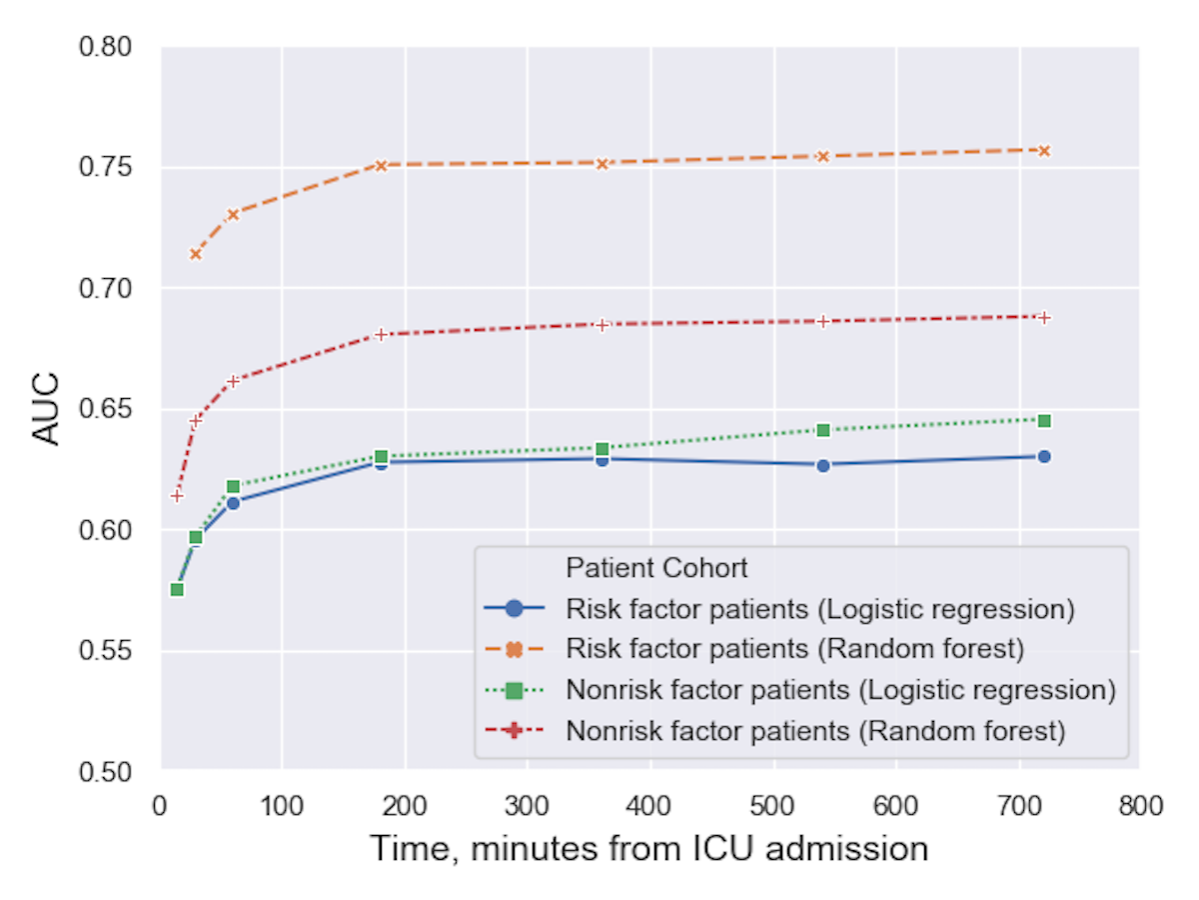
Predication AUC for risk factor and nonrisk factor patients with variable vital signs extraction time windows from 15 minutes to 720 minutes using only vital signs as model inputs. The x-axis represents the total number of minutes from ICU admission that vital signs were extracted from the database, meaning at higher time values more data was extracted. AUC: area under receiver operating characteristic curve; ICU: intensive care unit.

Using only aggregate vital signs as data inputs we evaluated model performance across variable vitals data extraction windows. Figure 4 illustrates AUC values (y-axis) of each model at different extraction window sizes (x-axis). In both models, there appears a point of diminishing returns around 200 minutes where additional vital signs data do not continue to improve results. This behavior is seen in both prediction models across all patient cohorts.

**Figure 5:**
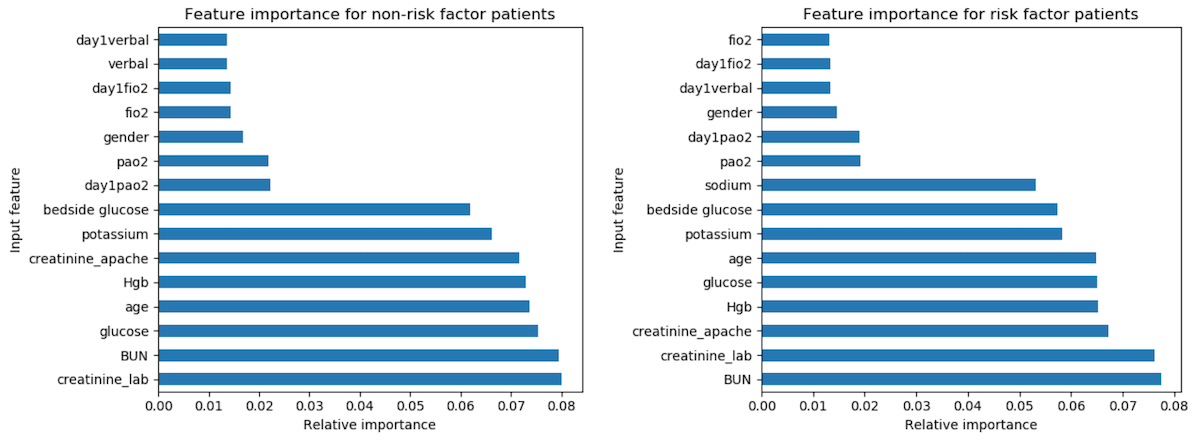
Random forest feature importance with 150 estimators for nonrisk factor and risk factor patients. BUN: blood urea nitrogen.

We then used the random forest model to identify which discrete features were most influential in predicting heart failure by plotting the relative feature importance. We applied the same number of estimators (n_estimators=150) and calculated feature importance for all lab values and APACHE variables (Figure 5). The selected top features were similar between *risk factor* and *nonrisk factor patients*. In addition, many of the top 10 features are laboratory values, even though, when used as individual inputs, APACHE variables outperformed laboratory measurements.

## Discussion

### Performance and Clinical Relevance

In this study, we evaluated two interpretable prediction models for decompensation in critical care using heart failure onset as a target outcome. Both logistic regression and random forest were evaluated as close to the time of ICU admission as possible using multiple types of input features.

We found that results across all four cohorts showed reasonable prediction accuracy. Generally, random forest outperformed multivariate logistic regression. On an individual basis, APACHE variables predicted heart failure onset better than laboratory measurements or vital signs; however, the best performance was achieved when model inputs were combined. Trials consisting of APACHE and laboratory measurements or all three data inputs (APACHE, labs, and vitals) had the highest performance metrics compared to any individual trial. This was corroborated by random forest feature selection highlighting several laboratory measurements as important to heart failure prediction relative to other input features.

Although vital signs near the time of ICU admission improve heart failure predictions when combined with other inputs, overall, vital signs results individually were not strong. Methodologically, vital signs and laboratory measurements, however, are promising for future prediction models. Traditional severity scoring models, such as APACHE, use data from only the first 24 hours of an ICU stay. Laboratory measurements and vital signs, however, are typically monitored on a continuous or semicontinuous basis throughout the length of an ICU stay. This would allow for future iterations of our prediction models to make predictions closer to the time of heart failure rather than being limited to ICU admission time. The continuous monitoring of vital signs and temporal value of laboratory measurements could also allow predictions to be made prospectively on a semicontinuous basis (eg, prediction output every 3 hours).

In addition, vital signs AUC values in Figure 4 suggest that there is an optimal threshold in the size of data extraction window for both predictive performance and computational load, and could inform future prediction models. If not enough data are extracted, results are diminished. Similarly, a data extraction time window too large increases computational load and does not necessarily improve performance.

Prediction window variation has been applied over longer time periods and multiple hospital visits for heart failure detection. We applied a similar methodology over a much shorter time frame more appropriate for ICU visits. Earlier predictions allow clinicians to determine patient prognosis and begin appropriate intervention. Clinicians may also revisit disease state predictions throughout a patient stay based on treatments or emergence of comorbidities.

Higher frequency continuous vital signs data in conjunction with laboratory measurements are a feasible option for predicting heart failure or other patient decompensation events in critical care through tele-ICU data early in an ICU stay. Vital signs tend to be available upon admission and continue through the majority of a patient ICU stay allowing for semicontinuous predictions. Real-time predictions throughout a patient stay are particularly useful for illnesses such as heart failure where poor outcomes can range from chronic to acute onset. In addition, heart failure mode of death assessments illustrate high variability as well and require predictions that facilitate timely interventions specific to the associated risks [17].

Results were similar between *risk factor* and *nonrisk factor patients* meaning accurate heart failure prediction will likely be made for patients not presenting with an indication of apparent risk of heart failure. This is supported by the similar AUC, precision, recall, and F1 scores across both models for *nonrisk factor patients* and could be used to inform ICU clinicians of impending failure for patients not initially deemed at risk.

### Challenges and Limitations

The prediction models in this study demonstrate the viability of machine learning applications leveraging remote monitoring data to further alleviate the challenges imposed by complex and data-intensive critical care environments, and contribute to the prognostication of cardiovascular diseases in the ICU. Our prediction models, however, may be partially influenced by and do not compensate for potential bias due to ICD-9 coding practices. Heart failure is not an explicitly defined event but rather a patient state in which the heart is struggling to function properly and as such is difficult to diagnose.

Moreover, vital signs data were collected using bedside monitoring systems as 1-minute averages and archived into the database as 5-minute median values. This decreased granularity over varying time windows of vital signs data extraction. Data may miss critical, subclinical cardiovascular events. Additional information loss occurs by reducing vital signs from time series data to discrete aggregate values. Data collection frequencies, however, are generally dependent upon what measurements are being taken from each patient at the bedside and at what times during their ICU stay. This can also cause high variability in time intervals between data points for each patient unit stay and total length of each time series.

Lastly, our approach does not account for the temporal relationship between vital signs data extraction or laboratory measurements and the prediction event. In an attempt to predict patient decompensation soon after ICU admission our variable data window begins at time of admission regardless of when heart failure onset may have occurred. Similarly, laboratory measurements are taken throughout a patient ICU stay, yet we retained only the first measurement in the interest of early decompensation prediction. An alternative approach to data aggregation is time series analysis of continuous, more granular, and physiologic data. This is corroborated by a recent study that showed the importance of temporal relations in recurrent neural network model inputs and is a possible future avenue for this work [29].

### Future Work

Logistic regression and random forest methods were selected based on interpretability and previous critical care applications using similar data inputs [30]. Model inputs, however, were limited to discrete variables. Alternatively, handling vital signs data as time series model inputs without overaggregating may yield improved results. A sliding window approach with real time series data and more powerful machine learning methods would allow for subsequent predictions to be made well after admission and throughout a patient stay [31]. This alternative approach would address the temporal relationship between the decompensation event (heat failure onset) and the input data used to make the prediction.

Ongoing and future studies also include analysis and machine learning application to specific events, which contribute to risk of heart failure onset (eg, myocardial infarction and pulmonary embolism). The ability to predict and potentially prevent these distinct events may subsequently avoid patient decompensation rather than predicting heart failure itself. In conjunction with feature selection, events or physiologic features most relevant to heart failure onset in critical care could be refined, thus, improving results. Model inputs could also be altered such that the heart failure risk factors are used as additional inputs rather than using risk factors for cohort segregation.

There are many different ICU types including cardiac ICUs. Heart failure may be managed differently in different critical care settings. Further research in this area could give insight to heart failure management variation. Our modeling approach may alleviate variations across ICUs by acting as a support system for clinicians focused on diagnoses other than heart failure.

### Conclusions

Remotely monitored critical care data offers opportunity for machine learning applications and deeper analysis than what may be possible at the bedside. Handling of disparate clinical data sources, data cleaning, preprocessing, and leveraging machine learning techniques may take place remotely so as to not disrupt existing ICU workflow and to provide complex clinical decision support. Risk factors for patient decompensation, or clinical deterioration, are prevalent in tele-ICU data as are clinical features sufficient for clinically relevant patient decompensation predictions with interpretable machine learning methods. Both logistic regression and random forest models were able to identify appropriate input features and narrowed data extraction time windows and thresholds for computational limitations at roughly 200 minutes after ICU admission. Our approach validates the feasibility of identifying decompensation events and patient risk factors, and making predictions using dissimilar data from variable timelines. More powerful machine learning approaches beyond regression and ensemble methods with alteration of our data extraction time window approach to avoid data aggregation could yield improved results in predicting heart failure onset or other patient decompensation events in critical care, albeit at the expense of interpretability.

## Appendix

Multimedia Appendix 1 Vital signs measurements used in all trials where model input variables included vitals measurements.

Multimedia Appendix 2 Supplementary multivariate logistic regression and random forest area under receiver operating characteristic curve and precision-recall curve for three individual risk factor groups: coronary artery disease, hypertension, and myocardial infarction.

## Abbreviations

APACHE: Acute Physiology and Chronic Health Evaluation
AUC: area under the receiver operating characteristic curve
ICD-9: International Classification of Diseases version 9
ICU: intensive care unit
P-R: precision-recall
