# Supplementary material for: Decompensation in Critical Care: Early Prediction of Acute Heart Failure Onset
Source: JMIR Med Inform. 2020 Aug 7;8(8):e19892. doi: 10.2196/19892 (PMC7442938; doi:10.2196/19892)
Supplement: Multimedia Appendix 1 [file medinform_v8i8e19892_app1.pptx]

## Slide 1
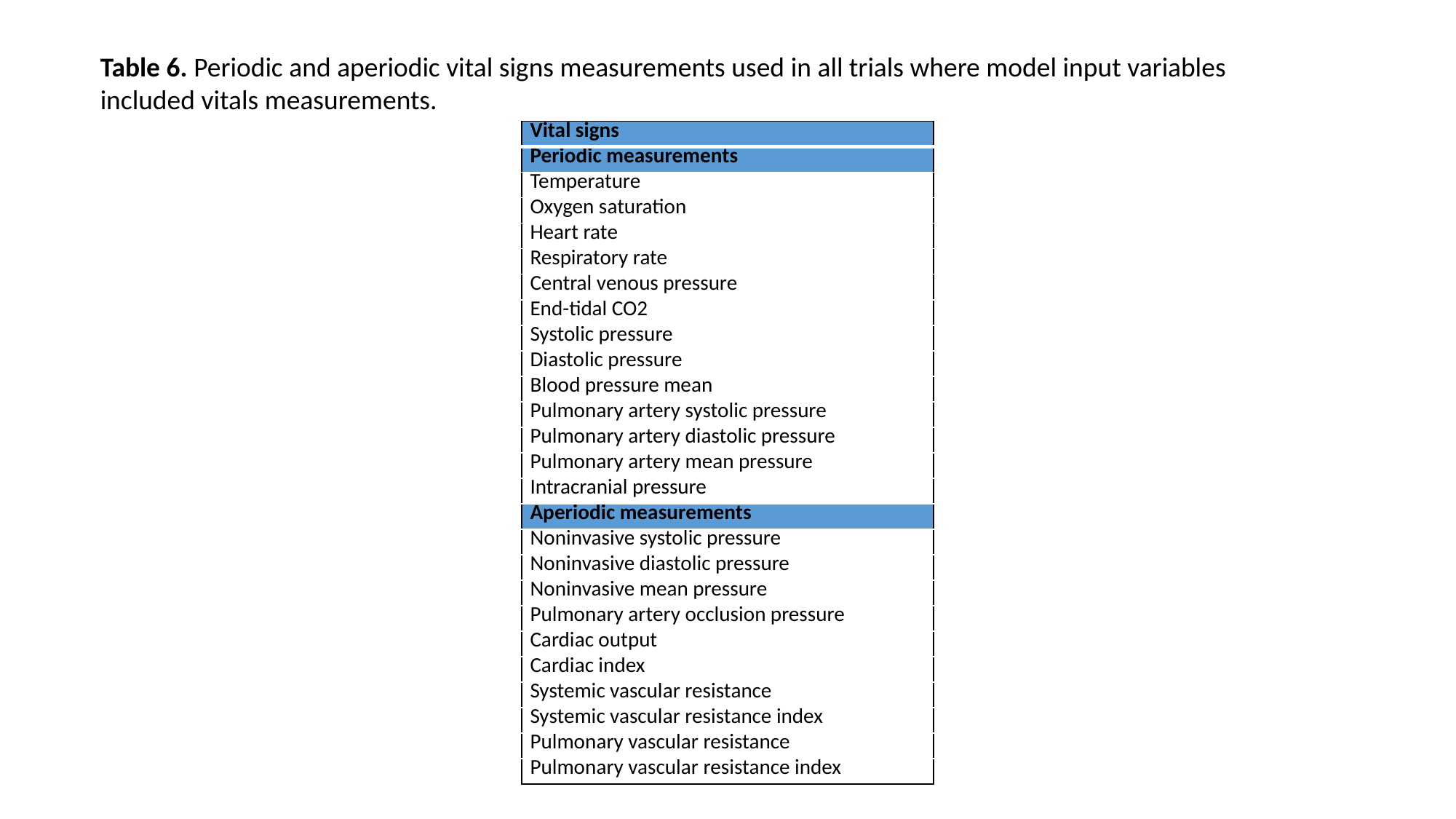

Table 6. Periodic and aperiodic vital signs measurements used in all trials where model input variables included vitals measurements.
| Vital signs |
| --- |
| Periodic measurements |
| Temperature |
| Oxygen saturation |
| Heart rate |
| Respiratory rate |
| Central venous pressure |
| End-tidal CO2 |
| Systolic pressure |
| Diastolic pressure |
| Blood pressure mean |
| Pulmonary artery systolic pressure |
| Pulmonary artery diastolic pressure |
| Pulmonary artery mean pressure |
| Intracranial pressure |
| Aperiodic measurements |
| Noninvasive systolic pressure |
| Noninvasive diastolic pressure |
| Noninvasive mean pressure |
| Pulmonary artery occlusion pressure |
| Cardiac output |
| Cardiac index |
| Systemic vascular resistance |
| Systemic vascular resistance index |
| Pulmonary vascular resistance |
| Pulmonary vascular resistance index |
