# Supplementary material for: Decompensation in Critical Care: Early Prediction of Acute Heart Failure Onset
Source: JMIR Med Inform. 2020 Aug 7;8(8):e19892. doi: 10.2196/19892 (PMC7442938; doi:10.2196/19892)
Supplement: Multimedia Appendix 2 [file medinform_v8i8e19892_app2.pptx]

## Slide 1
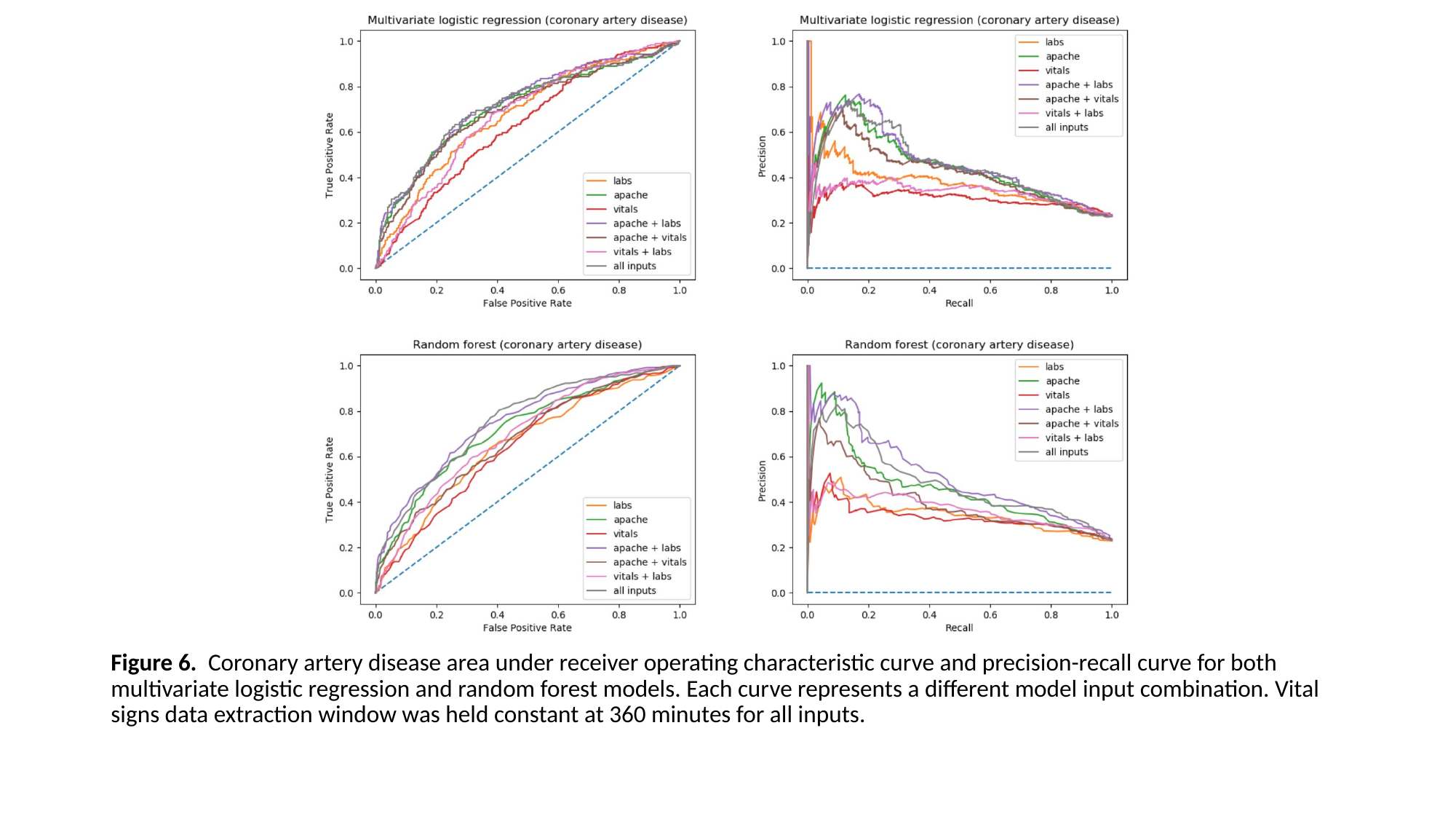

Figure 6. Coronary artery disease area under receiver operating characteristic curve and precision-recall curve for both multivariate logistic regression and random forest models. Each curve represents a different model input combination. Vital signs data extraction window was held constant at 360 minutes for all inputs.

## Slide 2
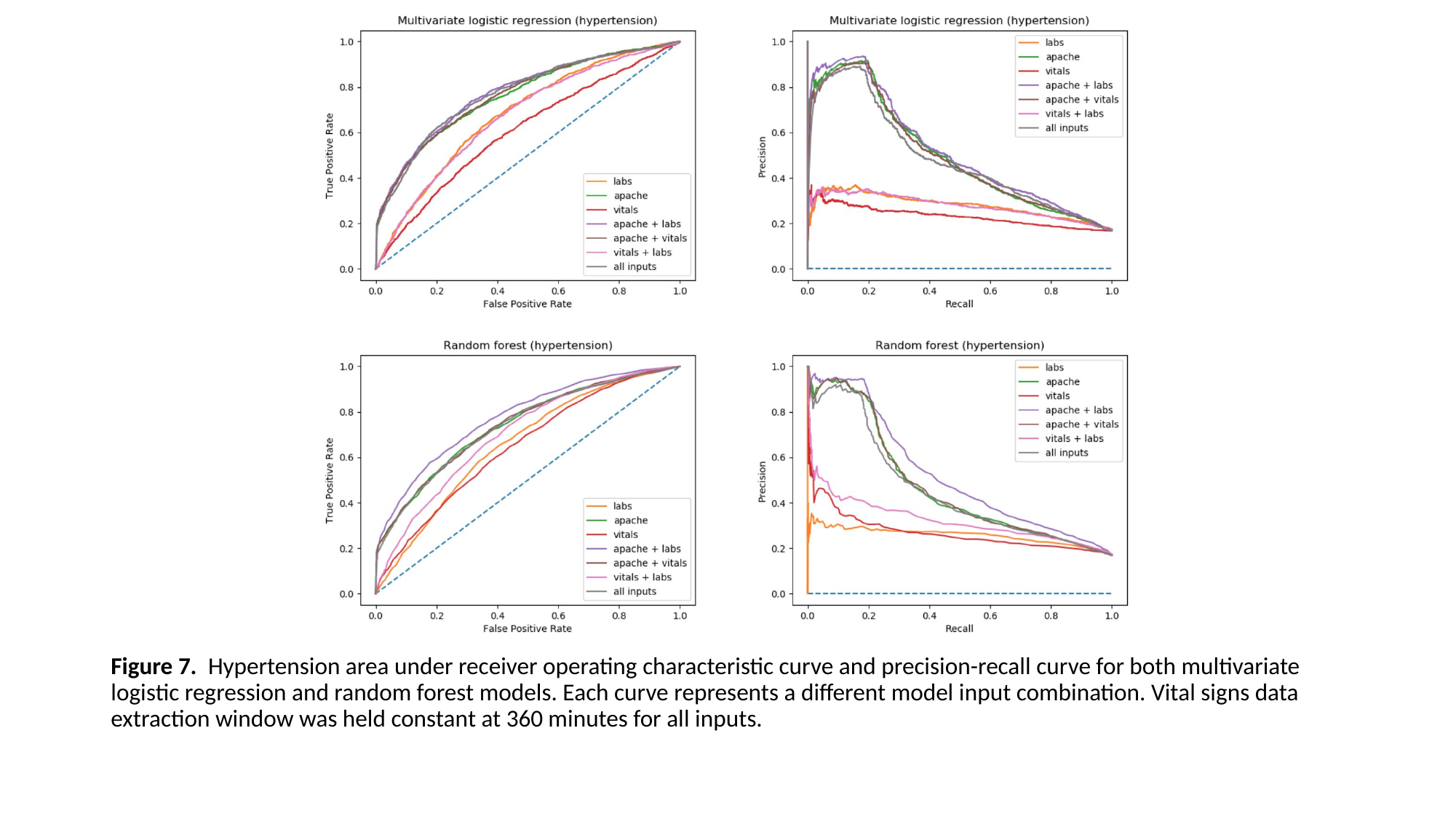

Figure 7. Hypertension area under receiver operating characteristic curve and precision-recall curve for both multivariate logistic regression and random forest models. Each curve represents a different model input combination. Vital signs data extraction window was held constant at 360 minutes for all inputs.

## Slide 3
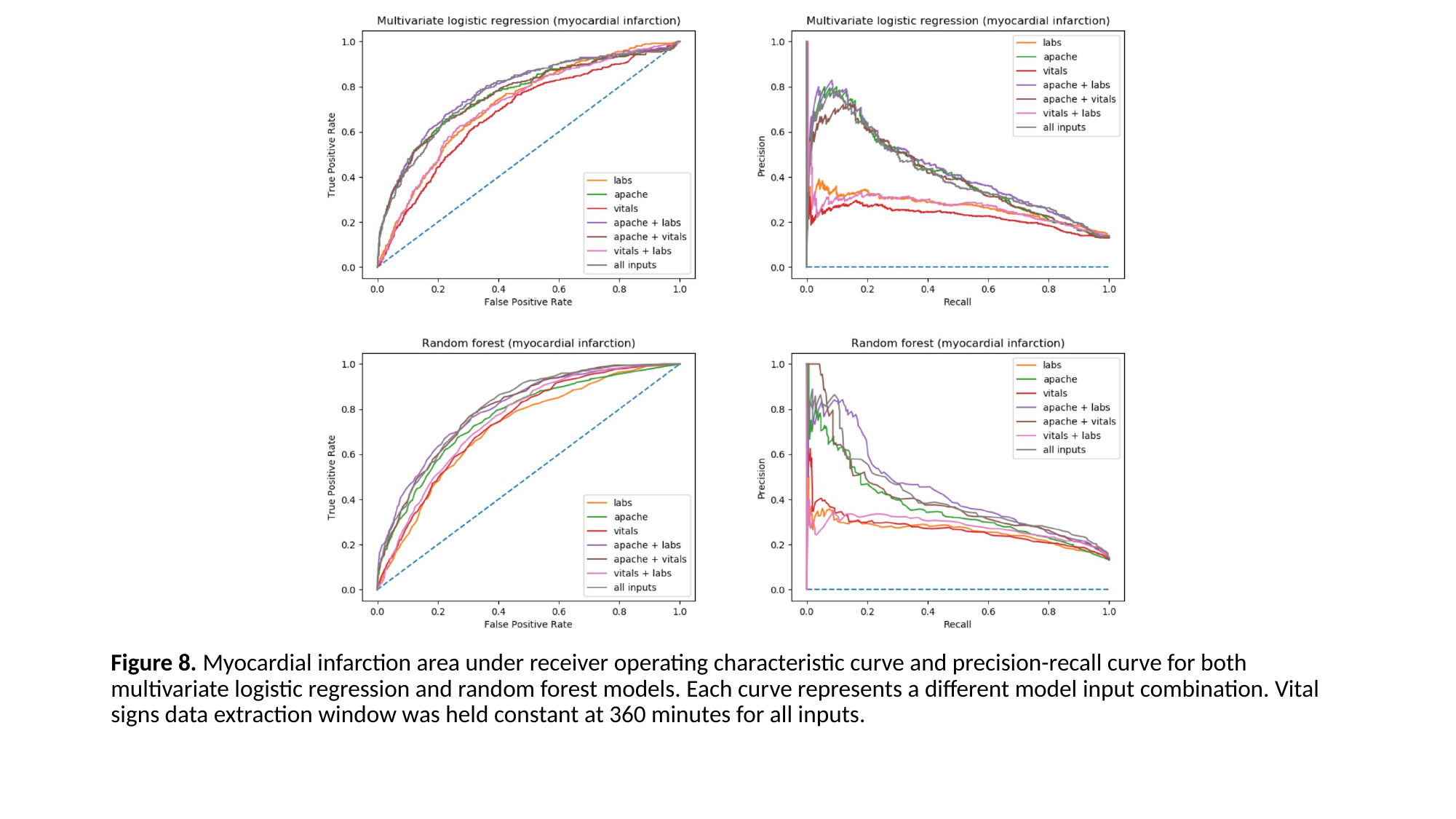

Figure 8. Myocardial infarction area under receiver operating characteristic curve and precision-recall curve for both multivariate logistic regression and random forest models. Each curve represents a different model input combination. Vital signs data extraction window was held constant at 360 minutes for all inputs.
